# Preliminary evidence that lectins in infant soy formula apparently bind bovine milk exosomes and prevent their absorption in healthy adults

**DOI:** 10.1186/s40795-022-00503-0

**Published:** 2022-01-21

**Authors:** Ezra Mutai, Alice Kah Hui Ngu, Janos Zempleni

**Affiliations:** Present address: Department of Nutrition and Health Sciences, University of Nebraska-Lincoln, 316C Leverton Hall, Lincoln, NE 68583 USA

**Keywords:** Bioavailability, Bovine milk exosomes, Human, Infant soy formula, Lectins, microRNA, Milk

## Abstract

**Background:**

Milk exosomes and their microRNA (miR) cargos are bioavailable. The content of exosomes and miRs is negligible in infant formulas compared to human milk, and dietary depletion of exosomes led to changes in bacterial communities and impaired gut health in juvenile mice. Adverse effects of formula feeding may be compounded by using soy formulas due to exosome binding by abundant lectins in that matrix. The purpose of this study was to assess the bioavailability of milk exosomes and their miR cargos added to soy formula in adults, as well as the potential role of soy lectins in exosome bioavailability.

**Methods:**

Eleven healthy adults (6 men, 5 women) enrolled in this randomized crossover study. Participants consumed 1.0 l of soy formula without (SF) or with (SFE) bovine milk exosomes added. Concentration-time curves of six plasma miRs were analyzed using reverse transcription quantitative PCR. Lectin affinity chromatography was used to assess the binding of exosomes by soy lectins. Data were analyzed by using paired *t* test. *P* < 0.05 was considered statistically significant.

**Results:**

Consumption of SF and SFE did not elicit postprandial increases in plasma miRs. Approximately 39% of bovine milk exosome particles were retained by lectin columns.

**Conclusions:**

We conclude that fortification of soy formulas with milk exosomes, in the absence of removing lectins, is not a viable strategy for delivering bioavailable exosomes and their miR cargos. Lectins in soy formulas bind glycoprotein on the surfaces of milk exosomes, thereby preventing exosome absorption.

**Trial registration:**

ISRCTN registry ID: 16329971. Retrospectively registered on February 7th, 2019.

## Introduction

Exosomes play essential role in cell-to-cell communication, which is facilitated by the binding of exosomes to receptors on the surface of recipient cells or internalization of exosomes and their cargos by recipient cells [1]. Among the regulatory cargos of exosomes, microRNAs (miRs) are of particular interest because more than 60% of human genes have conserved miR-binding sites, humans synthesize approximately 2000 miRs, and loss of miR maturation is embryonic lethal [2–5]. MiRs alter gene expression by binding to complementary sequences in the 3′-untranslated regions in mRNA, leading to mRNA degradation or blockage of translation [6, 7].

Exosomes are present in virtually all fluids, including human and bovine milk [8–11]. Encapsulation of miRs in exosomes confers protection against degradation by low pH and enzymes in the gastrointestinal tract [9] and a pathway for internalization by receptor cells [12]. We were the first to report that exosomes and their miR cargos may not solely originate in endogenous synthesis but may also be absorbed from milk [13–15]. Our discoveries were confirmed by independent laboratories while a few laboratories raised the concern that the amount of miRs absorbed from milk may be insufficient to elicit biological effects (reviewed in [16]).

Dietary depletion of milk exosomes and their RNA cargos elicited phenotypes such as increase in purine metabolites in human and murine body fluids and tissues [17]. Dietary depletion of bovine milk exosomes (BMEs) caused a variety of phenotypes in mice including a moderate loss of grip strength, an increase in the severity of symptoms of inflammatory bowel disease, a decrease in postnatal survival and changes in bacterial communities in the ceca [17–21], whereas BMEs enhanced goblet cell activity and prevented the development of necrotizing enterocolitis in mice [22]. These observations raise concerns regarding infant nutrition by using formulas, because previous reports agree that the content of exosomes and miR cargos is modest to not detectable in milk formulas [9, 11, 23]. The nutritional importance of these observations has been highlighted in a recent review [24]. Effects may be compounded in infants fed soy formulas, which lack milk altogether. The global soy infant formula market is predicted to grow $278 million during 2019 to 2023 [25]. One might want to explore the possibility to fortify soy formulas with milk exosomes to make them more similar to human milk. There is precedent for optimizing nutrient content of infant formulas through fortification by formula manufacturers [26, 27]. The mere fortification of soy formulas with milk exosomes could prove problematic because lectins, including those in soy, bind to carbohydrates and aggregate cells and exosomes [28, 29]. In soybeans, 0.5% of proteins are lectins [30]. Proteins on the outer exosome surface are extensively glycosylated [31], making the targets for precipitation by lectins. Lectins in legumes such as soy are resistant to heat denaturation, and heating of legumes at 70 °C for several hours has little or no effect on lectin activity [32]. Infant formula is pasteurized by holding a temperature of up to 94 °C for up to 30 s [33].

This study assessed the bioavailability of exosomal miRs in soy infant formulas fortified with BMEs. Because of the need to perform serial blood collections, this study was conducted in healthy adults as a model for infants. We focused on immune-related miRs (miR-15b, miR-21-5p, miR-34a-5p, miR-106b, miR-155, and miR-223), because they are abundant in human milk and their nucleotide sequences are identical in humans and cows [3, 34].

## Methods

### Participants

Eleven healthy adults (6 men, 5 women) participated in this study. Subjects were 28.8 ± 3.51 years old and had a BMI of 23.6 ± 2.24 kg/m^2^ (means ± SD). Exclusion criteria included pregnancy, smoking, milk allergies, and self-reported health problems. The Institutional Review Board at the University of Nebraska-Lincoln approved this protocol, and all participants provided a signed informed consent form before participation (protocol number IRB# 20131013755FB). This study was retrospectively registered as a clinical trial with the ISCRTN registry (ISRCTN16329971) on 07/02/2019.

### Experimental design of the formula feeding study

We used a randomized cross-over design. Participants were randomly assigned to treatment groups (Enfamil; milk-free and lactose-free powder with iron) without (SF) or with (SFE) BME fortification and a washout period of at least 1 week between treatments by using computerized numbers. Bovine milk contains approximately 10^14^ exosomes per milliliter [35]. Exosomes were isolated from 1.0 l bovine milk (1% fat) obtained from a local grocery store. Exosomes were isolated by ultracentrifugation as described previously [14, 36] and re-suspended in a final volume of 1 ml in sterile phosphate-buffered saline and stored at − 80 °C until use. In previous studies we reported the results from BME authentication by transmission electron microscopy, Nanosight size analysis and immunoblotting of exosome markers and markers of extracellular vesicles other than exosomes [14, 15, 35]. Soy infant formula (Enfamil; milk-free and lactose-free powder with iron) was purchased from a local grocery store. A 1.0-l dose of soy infant formula was prepared following the manufacturer’s instructions. For fortification of soy formula drinks, a homogenous suspension of 1 ml of exosomes obtained from 1.0 l of bovine milk was added to the soy infant formula drink, and thoroughly mixed before human consumption. We assessed the miR content in BMEs by RNA-sequencing analysis and reported the findings in a previous publication [35] A 28-yr-old male participant (77 kg body weight, 1.72 m height) served as reference and consumed 1.0 l formula. The volume consumed by the other subjects was adjusted by total body water [37]. The volume consumed equaled 0.84 ± 0.15 l (mean ± SD) for the 11 participants. This dose produced a robust increase in plasma miR concentrations in a previous study in a similar cohort of adults [13]. Participants consumed SF or SFE in a randomized order.

### Sample collection and miR analysis

Blood samples were collected before (baseline; time = 0 h) and 3, 6, and 9 h after formula consumption. Blood samples were collected in EDTA tubes to avoid loss of exosomes by binding to heparin [38]. Plasma was separated from cellular materials by using Histopaque gradient centrifugation and stored at − 80 °C until miR analysis [39]. Samples compromised by hemolysis were discarded, because the release of miRs from red blood cells is a confounder in miR analysis [40]. The nucleotide sequences of the vast majority of mature bovine miRs are identical to their human orthologs, including miR-1, miR-15b, miR-21-5p, miR-34a-5p, miR-106b, miR-155 and miR-223 [3]. MiR-1 was tested because it is not detectable in bovine milk exosomes (negative control). The other six miRs were tested because they were the focus of a parallel study of the amelioration inflammatory bowel disease by miRs in milk exosomes [19]. Note that some of these miRs are less abundant than miR-30d, miR-148a and the let-7 family of miRs in human and bovine milk exosomes [9, 11, 24]. A synthetic miR, miSPIKE (IDT DNA, Inc.) was added to plasma samples after denaturation with lysis buffer and served as external standard. Plasma miRs were measured by using reverse transcription quantitative PCR (RT-qPCR) as previously described, modified by purification of columns in NucleoSpin miRNA plasma kit (Macherey-Nagel) described below [13]. The kit provides a proprietary universal reverse primer, whereas forward primers are miR specific (Table 1). Under the experimental conditions, miRs that produced Ct values ≤30.0 were considered detectable. Concerns were raised regarding the contamination of spin columns for RNA isolation with microbial RNAs, which might cause artifacts in miR analysis [41]. We formally tested for column contamination by passing molecular biology grade water through hypochlorite-treated and non-treated columns and compared the Ct values of the six miRs in the two treatments by RT-qPCR. Although, we could not reproduce the finding that spin columns are contaminated (see Results), we decided to err on the side of caution and treated columns with 0.5% sodium hypochlorite [41].

**Table 1:** PCR primers used for the quantification of miRs in human plasma

Amplicon	Forward Primer
miSPIKE	CTCAGGATGGCGGAGCGGTCT
miR-1	TGGAATGTAAAGAAGTATGTAT
miR-15b	TAGCACATCATGGTTTACA
miR-34a-5p	TGGCAGTGTCTTAGCTGGTTGT
miR-106b	TAAAGTGCTGACAGTGCAGAT
miR-155	TTAATGCTAATCGTGATAGGGGT
miR-21-5p	GCTAGCTTATCAGACTGATGTTGA
miR-223	CTGTCAGTTTGTCAAATACCCCA

### Lectin affinity chromatography

Plastic syringes (3-ml volume; BD Biosciences cat. no. 309657) were fitted with Grade 6 qualitative filter paper (Whatman cat. no. 1006–125) in the bottom and packed with 2 ml agarose-bound soybean agglutinin (Vector Laboratories, cat. no. AL-1013) and equilibrated with binding buffer (10 mM HEPES, 150 mM NaCl, pH 7.5) at room temperature. A 1-ml suspension of BMEs with particle and protein concentrations (10^13^ BMEs; 19.8 mg/ml protein) was loaded and 1 ml flow through (referred to as FB in Results) was collected. One 1 ml binding buffer was added, the stopcock was closed, and columns were incubated at room temperature for 60 min. Columns were washed with binding buffer and 1-ml fractions were collected until the absorbance at 280 nm in the eluate returned to baseline values (referred to as F1- F16 in Results). Next, elution buffer (10 mM HEPES, 150 mM NaCl, 200 mM N-acetylgalactosamine, pH 7.5) was applied and 100-μl fractions were collected (referred to as E1- E30 in Results). The absorbance at 280 nm in fraction E30 was the same as in elution buffer (blank). The number of BMEs in F1 – E30 was assessed by using a Malvern NanoSight NS300 nanoparticle tracking instrument.

### Statistical analyses

Normality of data distribution was determined by using the Kolmogorov-Smirnov test. Data were analyzed by using paired *t* test. Data were calculated by using GraphPad Prism 6 (GraphPad Software). Data are reported as means ± SD. Differences were considered statistically significant if *P* < 0.05.

## Results

### Bioavailability of miRs in SF and SFE

Four of the six miRs tested were detectable in human plasma, miR-15b, miR-21-5p, miR-106b and miR-223. MiR-34a-5p and miR-155 were not detectable before and after consumption of SF and SFE (Ct values ≈34). Both SF and SFE failed to elicit a significant postprandial increase in plasma miR concentrations for any of the miRs tested (Table 2). MiR-1 was used as a negative control because it is not detectable in bovine milk [13]. The Ct values for miSpike were not significantly different among time points but were well within detection limits (Ct values 22 ± 1.1 to 23 ± 1.9), whereas Ct values were 31 ± 1.3 to 33 ± 1.9 for miR-1. We could not reproduce a previous report that suggested contamination of spin columns with small bacterial RNAs. Briefly, when we passed molecular biology grade water through hypochlorite-treated and non-treated columns and compared the Ct values of the six miRs in the two treatments by RT-qPCR (*N* = 5 per treatment), Ct values were greater than 35 in all samples tested.

**Table 2:** C_t_ values of plasma miRs before and after consumption of SF and SFE in healthy adults^1^

C_**t**_ values								
	**miR-15b**		**miR-21-5p**		**miR-106b**		**miR-223**	
**Hour**	SF	SFE	SF	SFE	SF	SFE	SF	SFE
**0**	30 ± 0.9	30 ± 0.8	29 ± 1.7	30 ± 1.3	31 ± 1.4	32 ± 1.1	30 ± 2.2	30 ± 1.9
**3**	30 ± 0.8	30 ± 1.0	29 ± 1.6	30 ± 1.6	30 ± 1.2	31 ± 1.3	29 ± 1.5	29 ± 1.8
**6**	30 ± 1.1	30 ± 1.8	29 ± 1.5	29 ± 2.1	31 ± 1.5	31 ± 1.9	29 ± 1.7	30 ± 2.3
**9**	30 ± 1.2	30 ± 2.0	29 ± 1.6	29 ± 1.9	31 ± 1.0	32 ± 1.3	30 ± 2.2	30 ± 2.6

^1^Values are means ± SD, *n* = 11, *P* > 0.05 vs hour 0

*Ct* cycle threshold, *miR* microRNA, *SF* soy formula, *SFE* soy formula fortified with bovine milk exosomes

**Fig. 1:**
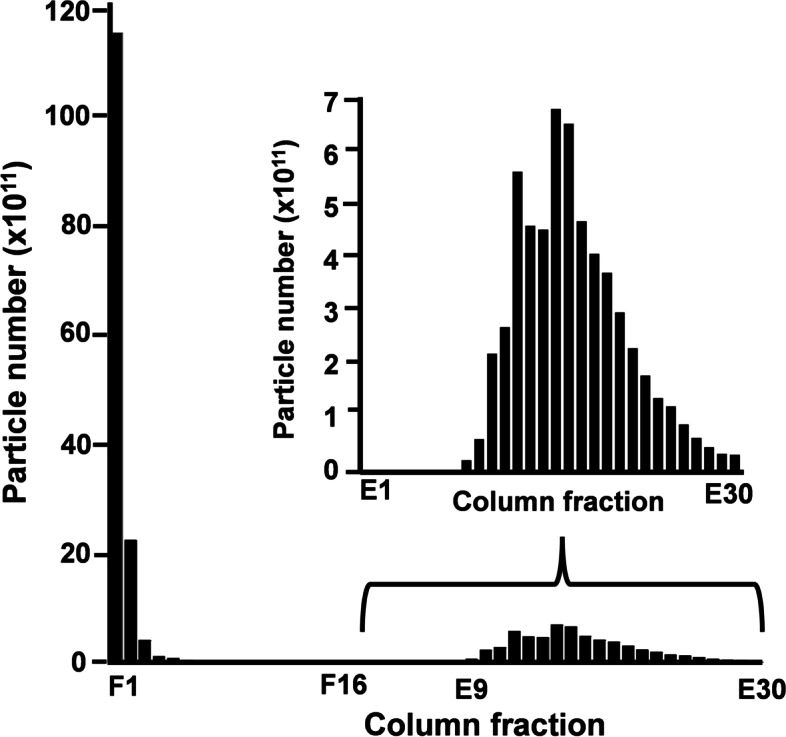
Retention of bovine milk exosomes by lectin affinity chromatography. Fractions F1 – F16 were collected after loading and elution with 1 ml binding buffer; fractions E1 – E30 were collected after elution with 1 ml elution buffer

The failure of BMEs fortification to elicit a postprandial increase in plasma miR concentrations is consistent with agglutination of BMEs by the soy lectins in formulas. We formally tested for this possibility by using soy lectin affinity chromatography. Approximately 39% from a suspension of BMEs in 1.4 × 10^13^ particles/buffer were retained by agarose-bound soybean agglutinin (Fig. 1), suggesting that the addition of BMEs to soy formulas is not a viable fortification strategy.

## Discussion

This study represents an important advance in the field of milk exosomes and their miR cargos, particularly as it relates to infant nutrition. SF does not contain milk exosomes and the mere addition of BMEs in SFE does not lead to a detectable increase in the plasma concentrations of the six miRs tested here. Our studies provide evidence that absorption of BMEs added to SF is prevented by BMEs agglutination by soy lectins. While the importance of dietary miRs for the optimal development of infants remains to be demonstrated, evidence suggests that milk exosomes may have positive effects on the health of infants. It remains to be determined whether removal of lectins from SFs can be achieved on a large scale at reasonable cost, and whether such treatment is desirable from a dietary point of view. Note that, in addition to the studies of SFs reported here, milk formulas also contain amounts of exosomes and miRs far below those in human milk [9, 11, 23].

Most Americans do not meet the recommendation by the American Academy of Pediatrics to exclusively breast feed infants in the first 6 months of life [42]. Only 26% of the children born in 2017 were exclusively breastfed through 6 months in the U.S. [43]. When framed in the context of the number of births in the U.S., approximately 2.78 million out of the 3.75 million infants born in the U.S. in 2019 were exclusively or partially fed with formulas during the first 6 months of life, i.e., the magnitude of translational activities must not be underestimated [44]. It has been reported that infants fed with soy formulas displayed transient delays in cognitive performance compared to breastfed infants, and formula-fed infants have a greater risk for becoming obese and develop diabetes and cardiovascular disease later in life [45–51].

Our findings point toward additional studies needed in the field of milk exosomes and infant nutrition. One could consider identifying the glycan features on the exosome surface responsible for the interaction between bovine exosomes and human cells. For example, future studies need to assess whether depletion of lectins in soy formulas results in the absorption of BMEs and their miR cargos added to formulas and explore the removal of lectins under controlled conditions. Ongoing studies in our laboratory address this question, as well as non-canonical pathways of exosome signaling such as changes in the gut microbiome, the binding of RNAs to Toll-like receptors, and biological activities of RNAs other than miRs. Finally, we intend to assess the developmental advantage, if any, conferred by milk exosomes to nursing offspring in mammals.

## Conclusions

We conclude that fortification of SF with BMEs is not a viable strategy when the goal is to provide bioavailable exosomes and miR cargos to infants. Any such strategies would need to be accompanied by prior removal of lectins from soy formulas.

## Data Availability

All data generated during this study are included in this published article.

## Abbreviations

BMEs: Bovine milk exosomes
Ct: Cycle threshold
miR: microRNA
RT-qPCR: Reverse-transcription quantitative PCR
SF: Soy formula
SFE: Soy formula fortified with bovine milk exosomes
